# Treatment of latent *Mycobacterium tuberculosis* infection with 12 once weekly directly-observed doses of isoniazid and rifapentine among persons experiencing homelessness

**DOI:** 10.1371/journal.pone.0213524

**Published:** 2019-03-13

**Authors:** Nwabunie Nwana, Suzanne M. Marks, Edward Lan, Alicia H. Chang, Michael Holcombe, Sapna Bamrah Morris

**Affiliations:** 1 ORISE Research Participation Program at Division of Tuberculosis Elimination, National Center for HIV/AIDS, Viral Hepatitis, STD and TB Prevention, Centers for Disease Control and Prevention, Atlanta, Georgia, United States of America; 2 Division of Tuberculosis Elimination, National Center for HIV/AIDS, Viral Hepatitis, STD and TB Prevention, Centers for Disease Control and Prevention, Atlanta, Georgia, United States of America; 3 Los Angeles County Department of Public Health Tuberculosis Control Program, Los Angeles, California, United States of America; 4 Mississippi State Department of Health Tuberculosis Program, Jackson, Mississippi, United States of America; University of Cape Town, SOUTH AFRICA

## Abstract

**Objectives:**

To investigate treatment outcomes and associated characteristics of persons experiencing homelessness who received 12-weekly doses of directly observed isoniazid and rifapentine (3HP/DOT) treatment for latent TB infection (LTBI).

**Methods:**

Among homeless persons treated with 3HP/DOT during July 2011 –June 2015 in 11 U.S. TB programs, we conducted descriptive analyses of observational data, and identified associations between sociodemographic factors and treatment outcomes. Qualitative interviews were conducted to understand programmatic experiences.

**Results:**

Of 393 persons experiencing homelessness (median age: 50 years; range: 13–74 years), 301 (76.6%) completed treatment, 55 (14.0%) were lost to follow-up, 18 (4.6%) stopped because of an adverse event (AE), and 19 (4.8%) stopped after relocations or refusing treatment. Eighty-one (20.6%) had at least one AE. Persons aged ≥65 were more likely to discontinue treatment than persons aged 31–44 years. Programs reported difficulty in following up with persons experiencing homelessness because of relocations, mistrust, and alcohol or drug use.

**Conclusions:**

This study demonstrates the feasibility of administering the 3HP/DOT LTBI regimen to persons experiencing homelessness, a high-risk population.

## Introduction

Approximately one quarter of the world’s population is infected with *Mycobacterium tuberculosis* without evidence of active TB disease, a condition known as latent tuberculosis infection (LTBI). Reactivation of LTBI, compared with rapid progression from recent infection, is estimated to account for greater than 80% of new TB cases in the United States.[1, 2]

Marginalized populations, including persons who live in or have lived in congregate settings (e.g., homeless shelters and correctional facilities), have a higher risk of exposure to *M*. *tuberculosis*.[3] In the United States, persons with a recent history of homelessness have an estimated TB disease incidence rate of 36–47/100,000 population [4] and have been documented to have more comorbid conditions than other TB patients.[5] Approximately 5% of TB cases in the United States are among persons who reported being homeless sometime within the 12 months preceding diagnosis.[2, 6] Studies of persons experiencing homelessness have reported higher rates of LTBI prevalence (20%)[7, 8] than that (5%) among the general US population.[9]

Targeted LTBI testing and treatment for populations experiencing homelessness is included as an important US strategy for TB elimination.[10] One of the greatest challenges to the success of LTBI treatment programs is inadequate patient adherence.[9, 11] As a result of inadequate adherence, the heretofore standard LTBI therapy of 9 months of isoniazid (9H) has completion rates under routine conditions of ≤50% and much less among persons experiencing homelessness.[12–14] The long treatment duration for 9H is reported to be a major reason for lack of patient adherence.[15, 16] Additionally, concerns regarding hepatotoxicity exist with 9H.[15]

In 2011, the U.S. Centers for Disease Control and Prevention (CDC) recommended use of a short-course regimen of 12 once-weekly directly-observed doses of rifapentine and isoniazid (3HP/DOT) for LTBI.[17] The PREVENT-TB clinical trial provided evidence of higher treatment completion with 3HP/DOT (82%), compared with 9 months of self-administered isoniazid (69%) (*P* < .01); the trial also determined that 3HP/DOT was non-inferior to 9H when comparing efficacy.[18] An evaluation under typical programmatic conditions documented the substantial improvement in treatment completion with 3HP/DOT over 9H, and provided some of the first data on 3HP/DOT treatment completion among persons experiencing homelessness.[19] However, additional data were needed to help describe factors related to patient adherence and the regimen’s acceptability among those experiencing homelessness.

In this study, we investigated treatment discontinuation rates and associated characteristics of persons experiencing homelessness who received 3HP/DOT as LTBI treatment in programmatic settings. We also assessed programmatic barriers and challenges to implementing use of 3HP/DOT among persons experiencing homelessness.

## Methods

This study used a subset of data from a previously published larger study [19] from a cohort of 3,288 eligible patients with LTBI who had been followed prospectively from initiation through completion of 3HP/DOT treatment at 16 TB program sites in the United States during July 2011–December 2013. Patients eligible for inclusion in this sub-analysis were 181 clinic patients at 10 program sites who were reported as having been homeless during ≤12 months before TB evaluation and as having received ≥1 dose of 3HP/DOT. An additional cohort of 212 persons experiencing homelessness (starting treatment between March 2013 and June 2015) was solicited from a U.S. site using similar processes for collection of data on the same variables. The additional site initiated the use of 3HP/DOT among persons experiencing homelessness after noting a worsening incidence of TB among persons experiencing homelessness in their community. Exclusion and inclusion criteria across sites were consistent with CDC guidelines[17] regardless of start date, with the exceptions of one site having an increased age-threshold in their decision to only treat patients aged ≥18 years (instead of ≥12 years of age) and another site having an added criterion that limited treating patients with liver function enzymes >3 times the upper limit of normal. For LTBI diagnostic testing, the majority of sites used QuantiFERON-TB Gold In-Tube Test (Quest Diagnostics, Secaucus, New Jersey) interferon gamma release assays (IGRA) to limit loss of patients observed in the additional clinic visit needed for tuberculin skin test (TST) results; one site used TST primarily.

Although program sites were not selected randomly, they were geographically distributed across the United States. Patients were considered ineligible if they had current active TB disease, were contacts of an index-patient with isoniazid- or rifampin-resistant TB, had a negative test for LTBI (tuberculin skin test [TST] or interferon-gamma release assay [IGRA]) at the time of TB evaluation, became pregnant during LTBI treatment, or were human immunodeficiency virus (HIV)-infected and on antiretroviral medications.[17] The definition of homelessness was consistent with that used by the U.S. Department of Housing and Urban Development developed in the original McKinney-Vento Act of 1987 and reestablished in the Hearth Act amendment to the McKinney-Vento Act of 2012; a homeless individual is defined as one “who lacks a fixed nighttime residence, whose primary residence is a temporary shelter, or whose primary nighttime residence that is a public or private place not designed for or ordinarily used as a regular sleeping accommodation for human beings, including a car, park, abandoned building, bus or train station, airport, or camping ground.”[20]

Program sites were designated as being in basic (two sites), standard (four sites) or comprehensive (five sites) tiers, depending on the detail of data collected. All sites collected the following patient information: demographics, weekly dose and symptom monitoring for adverse reactions to the regimen at each directly observed treatment (DOT) visit, baseline and monthly laboratory monitoring, and final disposition. Sites in the standard and comprehensive tiers collected additional information regarding medical or behavioral risk factors and concurrent medications during 3HP/DOT treatment.

### Quantitative data collection

Quantitative data from 11 sites were entered into a Microsoft Access (Microsoft Corporation, Redmond, Washington) database and analyzed by using SAS version 9.3 (SAS Institute Inc., Cary, North Carolina). Premature treatment discontinuation was defined as receipt of <11 doses of 3HP/DOT over 16 weeks. For descriptive statistics, we determined frequencies and percentages by patient characteristics. We also described frequencies and percentages of patients reporting adverse events (AEs) with and without treatment discontinuation. An AE was defined as any symptom reported since last receipt of a 3HP/DOT dose. Throughout treatment, patients were monitored for symptoms including those common to rifapentine and isoniazid using an identified symptom review checklist.[19] At each weekly DOT visit or at any interim visit or telephone call, patients were asked to report any AE or symptoms experienced since administration of the prior week’s medication dose.

### Quantitative data analyses

Among persons experiencing homelessness, we conducted bivariate log-binomial analyses to assess independent associations between patient characteristics and treatment discontinuation. We reported relative risk (RR) values with their 95% confidence intervals (CIs). We considered *P* values of < .05 as statistically significant. We also examined the effect of factors associated with 3HP treatment discontinuation using two multivariable log-binomial models. Factors considered for initial inclusion in the models were chosen based on the statistically significant findings from our bivariate analyses and clinical judgement of our subject matter expert. For the first model, we looked at the effect of patient socio-demographic characteristics and treatment reason on 3HP treatment discontinuation (N = 393). The second model only included patients from sites that collected data on medical and behavioral risk factors (N = 329), and examined the effect of socio-demographic characteristics, treatment reason, medical and behavioral risk factors on 3HP treatment discontinuation (N = 329). In both multivariable models, associations between covariates and 3HP/DOT treatment discontinuation were considered significant at a level of *P* < .05.

### Qualitative data collection

Qualitative data were collected through in-depth interviews with health care workers from a convenience sample of three program sites that treated a substantial proportion of study patients experiencing homelessness. The interviews were conducted to elicit responses about the programmatic experience of using 3HP/DOT to treat LTBI and to gain a more thorough understanding of quantitative results.

Interview questions were developed by using a thematic approach, then divided into three broad themes. The first theme focused on program-related questions (*e*.*g*., 3HP/DOT implementation start date, cost implications and staffing requirements). The second theme focused on the process of 3HP/DOT implementation, with certain questions relating to use of incentives and DOT while administering 3HP/DOT as well as description of the 3HP/DOT implementation process. Finally, questions related to the 3HP/DOT implementation process elicited provider perceptions of 3HP/DOT by those involved in the cascade of care. (See Appendix A for the specific list of questions in each broad theme). Two interviews were conducted by telephone, and one was conducted in person. The interview responses were recorded, transcribed into a Microsoft Word document, reviewed with site representatives for accuracy of information, and then collated into a spreadsheet.

### Ethics approval

From a human subjects ethics approval perspective, CDC determined this project to be public health practice, not research. Each participating site obtained ethical review and approval for participation in accordance with local requirements.

## Results

### Quantitative data

A total of 393 persons experiencing homelessness and who had LTBI started treatment with 3HP/DOT (median age 50 years; range 13–74 years) (Table 1); 308 (78.4%) male; 236 (60.1%) non-Hispanic black, 88 (22.4%) Hispanic, 57 (14.5%) non-Hispanic white, 6 (1.5%) non-Hispanic Asian, and 6 (1.5%) other. Fifteen (3.8%) were born outside the United States, and 86 (21.9%) were recent contacts of persons with active TB disease. Of the 393, 301 (76.6%) completed treatment. Ninety-two (23.4%) discontinued treatment; 55 (14.0%) were lost to follow-up, 18 (4.6%) stopped because of an AE, and 19 (4.8%) stopped because of other reasons (moved or refused 3HP/DOT treatment) (Table 1). Eighty-one (20.6%) had ≥1 AE, but only 18 (4.6%) discontinued because of an AE.

**Table 1:** Characteristics of persons experiencing homelessness for 11 program sites from July 2011-June 2015.

Patient Characteristics (N = 393)	No. (%)
**Site**	
A	7 (1.8)
B	162 (41.2)
C	8 (2.0)
D	4 (1.0)
E	5 (1.3)
F	60 (15.3)
G	5 (1.3)
H	4 (1.0)
I	135 (34.4)
J	1 (0.3)
K	2 (0.5)
**Sex**	
Male	308 (78.4)
Female	85 (21.6)
**Age group (years)**	
2–17	2 (0.5)
18–30	42 (10.7)
31–44	84 (21.4)
45–64	249 (63.4)
≥65	16 (4.1)
**Race/ethnicity**	
Hispanic	88 (22.4)
Non-Hispanic white	57 (14.5)
Non-Hispanic black	236 (60.1)
Non-Hispanic asian	6 (1.5)
Other	6 (1.5)
**Treatment reason^a^**	
Contact of person with active TB disease	86 (21.9)
Converter^b^	26 (6.6)
Inmate at correctional institution during previous year	28 (7.1)
Non-US–born	15 (3.8)
**Medical condition (n = 329)^a^**	
Diabetes	25 (6.4)
Chronic renal disease or on dialysis	1 (0.3)
Non-HIV–immunocompromised	0 (0.0)
Hepatitis (A, B, or C)	10 (2.5)
Chronic lung disease	11 (2.8)
Mental health problem	39 (9.9)
Hypertension	52 (13.2)
Thyroid disorder	1 (0.3)
HIV infection-positive	7 (1.8)
**Behavioral risk factors (n = 329)^a^**	
Alcohol use	59 (15.0)
Current or past smoker	109 (27.7)
Injection-drug use	9 (2.3)
Non-injection–drug use	42 (10.7)
**Final Disposition**	
Stopped treatment	92 (23.4)
Stopped because of adverse event	18 (4.6)
Stopped because of lost to follow-up	55 (14.0)
Stopped because of other reasons	19 (4.8)

Abbreviations: HIV, human immunodeficiency virus; TB, tuberculosis.

^a^ Patients could have had multiple treatment reasons, medical conditions, or behavioral risk factors.

^b^ Person with baseline tuberculin skin test who has ≥10mm increase in induration, or positive interferon-gamma release assay (IGRA) conversion from a baseline negative TB test, within a 2-year period.

Among the 329 patients with more than baseline data, hypertension (13%) was most frequently reported as a preexisting medical condition, followed by mental health problems (10%), and diabetes (6%). Twenty-eight percent were current or past smokers; 15% reported alcohol use (defined as more than 2 drinks per day); and 11% were non-injection-drug users (Table 1). Most frequently reported symptoms with use of 3HP/DOT treatment were nausea (6%), fatigue (5%), and sore muscles or joints (4%) (Table 2). In bivariate analysis, only two variables were significantly associated with treatment discontinuation for any reason: In comparison with those age 31–44 years, those aged ≥65 years were more likely to discontinue treatment (RR = 2.1; CI = 1.13–3.91; *P* = 0.04630). Contacts of persons with active TB were less likely to discontinue treatment (RR = 0.34; CI = 0.17–0.67; *P* **=** .00020) (Table 3). No deaths were reported.

**Table 2:** Symptoms reported with a short-course regimen of 12 once-weekly directly observed doses of Rifapentine and Isoniazid (3HP) among persons experiencing homelessness in 11 program sites from July 2011- June 2015 (N = 393).

Symptom/reaction^a^	Patient Reports of Symptoms No. (%)	Patient Reports of Symptoms Where Treatment Was Not Held or Stopped No. (%)	Patient Reports of Symptoms Where Treatment Was Held or Stopped No. (%)	Patient Reports of Symptoms, Treatment Held or Stopped but Patient Completed Treatment No. (%)	Patient Reports of Symptoms, Treatment Held or Stopped and Patient Did Not Complete Treatment No. (%)
**Loss of appetite**	9 (2.3)	6 (1.5)	3 (0.8)	0 (0)	3 (100)
**Nausea/vomiting**	23 (5.9)	18 (4.6)	5 (1.3)	0 (0)	5 (100)
**Diarrhea**	11 (2.8)	9 (2.3)	2 (6.9)	1 (50.0)	1 (50.0)
**Rash/hives**	11 (2.8)	7 (1.8)	4 (1.0)	1 (25.0)	3 (75.0)
**Fever/chills**	8 (2.0)	4 (1.0)	4 (1.0)	0 (0)	4 (100)
**Sore muscles/joints**	15 (3.8)	10 (2.5)	5 (1.3)	0 (0)	5 (100)
**Numbness/tingling**	12 (3.1)	11 (2.8)	1 (0.3)	0 (0)	1 (100)
**Fatigue**	19 (4.8)	14 (3.6)	5 (1.3)	1 (20.0)	4 (80.0)
**Dizziness/fainting**	12 (3.1)	6 (1.5)	6 (1.5)	1 (16.7)	5 (83.3)
**Abdominal pain**	12 (3.1)	12 (3.1)	0 (0)	0 (0)	0 (0)
**Yellow eyes/skin**	1 (0.3)	1 (0.3)	0 (0)	0 (0)	0 (0)
**Headache**	7 (1.8)	6 (1.5)	1 (0.3)	0 (0)	1 (100)
**Other**	20 (5.1)	12 (3.1)	8 (2.0)	1 (12.5)	7 (87.5)

^a^ Patients could report more than one symptom, thus symptom reports were not mutually exclusive

**Table 3:** Characteristics associated with treatment discontinuation among persons experiencing homelessness in 11 US sites from July 2011- June 2015.

Characteristics (N = 393)	Homeless Persons Not Completing Treatment No. (%)	Homeless Persons Completing Treatment No. (%)	Unadjusted RR (95% CI)
**Final Disposition**	92 (23.4)	301 (76.6)	
**Sex**			
Male	70 (22.7)	238 (77.3)	0.88 (0.58–1.33)
Female	22 (25.9)	63 (74.1)	Ref.
**Median age, years (IQR)**	53 (24)	50 (15)	1.00 (0.98–1.01)
**Age groups (years)**			1.00 (0.98–1.01)
2–17	0 (0)	2 (100)	0.0 (0.00–0.00)
18–30	14 (33.3)	28 (66.7)	1.40 (0.79–2.49)
31–44	20 (23.8)	64 (76.2)	Ref.
45–64	50 (20.1)	199 (79.9)	0.84 (0.53–1.33)
≥65	8 (50.0)	8 (50.0)	**2.1 (1.13–3.91)**
**Race/ethnicity**			
Hispanic	22 (25.0)	66 (75.0)	1.58 (0.79–3.19)
Non-Hispanic white	9 (15.8)	48 (84.2)	Ref
Non-Hispanic black	56 (23.7)	180 (76.3)	1.50 (0.79–2.86))
Other	5 (45.5)	6 (54.5)	**2.88 (1.19–6.96)**
**Treatment reason^a^**			
Contact of person with active TB disease	8 (9.3)	78 (90.7)	**0.34 (0.17–0.67)**
Converter^b^	4 (15.4)	22 (84.6)	0.64 (0.26–1.61)
Inmate at correctional institution during previous year	5 (17.9)	23 (82.1)	0.75 (0.33–1.69)
Non-US-born	5 (33.3)	10 (66.7)	1.45 (0.69–3.03)
**Sub analyses (n = 329)**			
**Sex**			
Male	68 (27.0)	184 (73.0)	0.94 (0.63–1.42)
Female	22 (28.6)	55 (71.4)	
**Median age, yrs (IQR)**	90 (27.4)	239 (72.6)	
2–17	0 (0)	2 (100.0)	0.60 (0.047, 7.76)
18–30	14 (35.9)	25 (64.1)	1.31 (0.75–2.30)
31–44	20 (27.4)	53 (72.6)	Ref.
45–64	49 (24.5)	151 (75.5)	0.89 (0.57–1.40)
≥65	7 (46.7)	8 (53.3)	1.70 (0.88–3.29)
**Race/ethnicity**			
Hispanic	3 (4.2)	68 (95.8)	0.48 (0.10–2.25)
Non-Hispanic white	3 (8.8)	31 (91.2)	Ref.
Non-Hispanic black	11 (5.1)	203 (94.9)	0.58 (0.17–1.98)
Other	1 (10.0)	9 (90.0)	1.13 (0.13–9.73)
**Treatment reason^a^**			
Contact of person with active TB disease	6 (24.0)	19 (76.0)	0.87 (0.42–1.79)
Converter^a^	3 (23.1)	10 (76.9)	0.84 (0.31–2.30)
Inmate at correctional institution during previous year	5 (19.2)	21 (80.8)	0.69 (0.31–1.54)
Non-US–born	5 (45.5)	6 (54.6)	1.70 (0.87–3.33)
**Medical condition^a^**			
Diabetes	5 (20.0)	20 (80.0)	0.72 (0.32–1.60)
Chronic renal disease	0 (0)	1 (100.0)	0 (0)
Immunocompromised	0 (0)	8 (100.0)	0 (0)
Hepatitis (A, B, or C)	5 (50.0)	5 (50.0)	1.88 (0.98–3.58)
Chronic lung disease	4 (36.4)	7 (63.6)	1.34 (0.60–3.0)
Mental health problems	12 (30.8)	27 (69.2)	1.14 (0.69–1.90)
Hypertension	13 (25.0)	39 (75.0)	0.90 (0.54–1.49)
**Behavioral risk factors^a^**			
Alcohol use	12 (20.3)	47 (79.7)	0.70 (0.41–1.21)
Current or past smoker	29 (26.6)	80 (73.4)	0.96 (0.66–1.40)
Injection drug use	3 (33.3)	6 (66.7)	1.22 (0.48–3.14)
Non-injection drug use	10 (23.8)	32 (76.2)	0.85 (0.48–1.51)

Abbreviations: CI, confidence interval; RR, risk ratio; IQR, interquartile range; TST, tuberculin skin test.

^a^ Patients could have had multiple treatment reasons, medical conditions, or behavioral risk factors.

^b^ Person with baseline tuberculin skin test who has ≥10mm increase in induration, or positive interferon-gamma release assay (IGRA)) conversion from a baseline negative TB test, within a 2-year period.

Reference group for risk ratios is persons without that characteristic, treatment reason, medical condition, or behavioral risk factor.

“Other” in race/ethnicity category includes Asian and other minor groups.

In the two final multivariable models, no factor was significantly associated with 3HP/DOT treatment discontinuation,—neither when controlling for the association of socio-demographic factors with 3HP/DOT treatment discontinuation nor when controlling for medical and behavioral factors.

### Qualitative data

Three sites (7 health care staff) participated in qualitative interviews. All three sites primarily targeted LTBI testing of persons experiencing homelessness, although one site tested additional populations. Staff perceived 3HP/DOT implementation cost to be high compared with standard 9H treatment at all three sites; nonetheless, staff understood that treating LTBI was a worthy investment for prevention of future, more-costly TB cases. Program staff did not receive additional funding for 3HP/DOT implementation; one program manager had convinced providers serving their homeless population outside of the health department setting to seek Medicaid reimbursement for 3HP/DOT. Only one site had hired additional staff (a nurse practitioner) to assist. All sites agreed that dedicated staff time or additional staff were required for expansion of services to contacts at homeless shelters. Sites used multiple resources and tools (*e*.*g*., informational brochures, personnel, and mental health or addiction services) to improve outreach to this population. Staff at one site emphasized using clear communication language throughout the entire spectrum of the LTBI testing and treatment cascade. As recommended, 3HP/DOT was administered by DOT at all sites; one site preferred administering medications in the clinic setting, whereas others preferred administering them at their local shelters.

Two sites provided incentives to patients, including food (*e*.*g*., $5 gift cards for fast-food restaurants) or transportation vouchers; one of these sites had a treatment completion rate of 98%. Site staff expressed personal beliefs that such incentives as bus tokens and gift cards were effective in increasing adherence when given to patients. Site staff did not make substantial changes to their protocols to accommodate treating persons experiencing homelessness.

Site staff were creative in finding external resources to support their LTBI treatment programs. Site managers collaborated with local entities during care delivery; one site partnered with shelter staff in providing transportation and targeted testing, and another partnered with correctional facilities for patients who became incarcerated during treatment. One consistent perception among staff was that they had limited experience with the 3HP/DOT regimen. Hospitals and laboratories also served as partners for completing the evaluations to rule out TB before initiating LTBI treatment; chest radiographs were frequently completed and interpreted at hospitals.

A challenge that programs encountered with treating persons experiencing homelessness was that continued instability in housing led to frequent patient relocation. Some programs reported encountering patients who mistrusted the regimen because it was characterized as new. The high percentage of persons using alcohol and drugs among this population also created additional challenges (such as frequent treatment interruptions) in administering and monitoring treatment.

Programmatic challenges mentioned by clinic staff included staffing and time requirements for treatment by DOT, patients’ difficulty with 3HP/DOT pill burden (three 300-mg pills/dose for isoniazid and six 150-mg pills/dose for rifapentine), higher costs of 3HP/DOT relative to 9H, and difficulty in following up with persons experiencing homelessness. Frequently reported AEs included high blood pressure while on 3HP/DOT medication. Previous and current substance users also reported concerns with triggering feelings or psychological feelings of being intoxicated (“high”) during blood draws.

All sites agreed that the short duration of 3HP/DOT held great promise for increased adherence and higher treatment completion rates needed for TB prevention among this population.

## Discussion

Although multivariable analyses did not yield significant medical, sociodemographic, or behavioral factors associated with treatment discontinuation, results from this study demonstrated that 3HP, a regimen of 12 doses of once-weekly rifapentine and isoniazid with DOT, was successful as a LTBI treatment option among persons who are reported as experiencing homelessness. The US Preventive Services Task Force recommends screening for and treating LTBI among persons in this high-risk population.[3] Compared with historic LTBI treatment completion using 6–12 months of isoniazid among homeless persons (25%–33%),[21] the treatment completion rate (77%) in our study was much higher.[18, 19] Given the frequent movement of this population, this high completion rate is difficult to achieve and is consistent with evidence in other populations that 3HP/DOT is positively associated with treatment completion.[18, 19, 22]

As elicited from the interview responses, factors contributing to the high completion were presumed to include short duration of the regimen, [22, 23] low rates of adverse events,[24] and use of incentives or enablers (food or transportation vouchers). A large-scale clinical trial (PREVENT TB) found greater rates of overall adverse reactions to 3HP compared with 9H, but lower rates of serious hepatotoxicity.[18, 25]

Past studies have documented that difficulty in treating LTBI among persons experiencing homelessness is principally a result of issues of instability (*e*.*g*., unstable housing, food insecurity, and high prevalence of mental health conditions),[26–29] and not necessarily a result of adverse events. Consistent with findings from these past studies, of 92 patients in our study who discontinued treatment, 55 (60%) discontinued because of being lost to follow-up. The rate of noncompletion of treatment resulting from reasons other than adverse events was consistent with the PREVENT TB clinical trial results. In that trial, the North American region reported that the proportion of discontinuation of LTBI treatment not associated with adverse events was 57% higher among participants with a history of homelessness than among participants with no such history (*P* < .001).[26, 30]

Our qualitative data collection efforts indicated that the relatively high treatment completion rate achieved with 3HP/DOT also may be attributed to the implementation process adopted by the programs. Two of the three interviewed programs (with treatment completion rates of 98% and 69%) administered treatment with incentives, ranging from food and transportation vouchers to gift cards. Past studies have demonstrated that targeted strategies among the homeless population (*e*.*g*., monetary incentives and education programs) improved adherence.[11, 31] Specifically, the timing of incentives (as elicited from site representatives) is important for adherence; an immediate monetary incentive is better than a deferred one.[31] In addition to incentives, administering treatment by DOT contributed to the level of success attained in treating this population. All three programs administered therapy by DOT, which has been documented to increase the likelihood that patients take their medications.[18, 31, 32] DOT delivered to patients where they were residing, such as in local shelter, also likely contributed to improved completion rates. CDC now also recommends use of 3HP by self-administration (SAT).[33] At U.S. sites, the iAdhere clinical trial found lower completion (77%) among all trial participants with self-administered 3HP compared with DOT administration (85%), but found the SAT administration to be non-inferior to that by DOT.[34] Among iAdhere trial participants who experienced homelessness, there was no significant difference in non-completion in adjusted analyses [34].

Project site staff mentioned that DOT, regardless of its effectiveness, contributed to the high cost of administering this therapy and might be an overall deterrent to wider adoption of 3HP under published guidelines that include administration by DOT.[17] Additionally, site representatives indicated that the high cost of rifapentine relative to that of isoniazid is another deterrent; however, since 2014, the price to TB programs of rifapentine was cut in half.[35] Regardless, the benefits of higher adherence to 3HP balances these upfront costs by increasing the number of TB cases prevented.[35] Studies of the cost effectiveness of 3HP (directly observed or self-administered) versus isoniazid in homeless populations are needed and are in progress. Lowering the rifapentine price and allowing 3HP self-administration might dramatically improve the cost-effectiveness of the 3HP regimen.[7, 35] The use of Video-DOT can also be leveraged as an alternative to in-person DOT for monitoring treatment. Furthermore, a fixed-dose 3HP pill might also positively impact treatment completion.

### Limitations

Given that this study was conducted under field conditions at programmatic sites, this paper includes certain limitations. First, the study was observational; self-selected sites implemented the project under their program’s routine practice, which was not standardized across sites. Given the observational design of this project, all persons experiencing homelessness and requiring LTBI treatment may have not been captured, leading to an underestimation of this cohort of patients. Possible limitations in interpreting completion rates might also exist because certain programs differed in instructions for stopping treatment as a result of an adverse event. Other limitations include underreporting of risk factors for low completion rates (*e*.*g*., substance abuse, mental health problems, or HIV infection status) because these factors were based on self-reporting. Lastly, the convenience sampling method used in selecting patients for the project may limit the generalizability of findings to other sites.

## Public health implications

This analysis demonstrated the feasibility of administering the 3HP/DOT LTBI regimen among persons experiencing homelessness, a population that is at high risk and has a high prevalence of behavioral risk factors that contribute to poor outcomes. This analysis demonstrated higher rates of treatment completion in this population than seen historically with the heretofore-standard 9H regimen. Use of 3HP as a shorter regimen appears to be conducive to completing treatment for LTBI and may play an important role in accelerating the decline of active TB disease in the United States by preventing future infectious TB cases among populations at risk.

## Appendix A

### Key informant interviews

Use of three months once-a-week dose of isoniazid and rifapentine (3HP) for latent tuberculosis infection (LTBI) among homeless persons in U.S. programmatic settings.

**Purpose of project**

The project is intended to assess and analyze factors contributing to and limiting treatment completion among homeless persons, and to eventually disseminate project findings through publication in a scientific journal.

The interview aims to elicit responses about your site’s programmatic experience surrounding the use of 3HP for treating homeless persons with LTBI. Other programs planning on using this regimen for treating homeless persons will benefit from the information gathered.

**Key informant interview questions**

**Program Questions**

1. When did your program start using 3HP for LTBI treatment (year, month, before or after guidelines)?
2. Was the use of 3HP targeted at a specific population(s); If so, which ones?
3. Did your program initially plan on treating homeless patients at the start of 3HP implementation?
4. What were the cost implications of treating homeless patients with 3HP?
5. How were project activities funded? Did you have to seek outside funding to support use of 3HP in the homeless population? Did you obtain any in-kind support for use of this regimen?
6. Did the 3HP program require more staffing to carry out protocol activities?
7. What resources and tools did you find useful to communicate effectively with homeless patients about taking 3HP?
8. What other resources and tools would be useful for clinics and health care providers on using 3HP with homeless patients?

**Process Questions**

9 Why did you decide to initiate use of 3HP for LTBI with homeless patients?
10 How did you determine or define if patients were homeless?
11 How did you determine if patients had LTBI? Which diagnostic tests were used?
12 What were your eligibility (inclusion and exclusion) criteria for treating a homeless patient for LTBI with 3HP?
13 Was the inclusion and exclusion criteria for homeless different from other populations?
14 Can you describe the overall process of 3HP implementation to target LTBI treatment among homeless patients?
15 Was treatment administered via DOT (directly observed therapy) or SAT (self-administered therapy), or both?
16 If DOT was used, was 3HP administered in clinical settings? Can you describe the settings (TB control program or public health only or FQHCs, Health Care for the Homeless Clinics)? Was 3HP provided to patients in other settings, like shelters or meal programs?
17 Was food provided to patients prior to or immediately after their dose?
18 Were any incentives provided? Can you describe the types of incentives used?
19 Did some incentives work better than others? If so, please describe which ones worked better and why you think that?
20 How were side effects monitored and assessed? Was this done differently than how it is routinely done in the clinic?
21 Was there a need to change site protocol to accommodate the use of 3HP among persons experiencing homelessness?
22 Were there partners in the delivery of care, and what was their role in the implementation process?

**Perception Questions**

23 What were the perceptions and expectations of using 3HP before your program started using it?
24 What do you find challenging about using 3HP in persons experiencing homelessness?
25 What were the positive experiences homeless patients expressed about taking 3HP?
26 What were the negative experiences homeless patients expressed about taking 3HP?
27 Was there a difference in the implementation of 3HP among homeless patients compared to stably-housed patients? If so, please describe.
28 Was there a difference in the implementation of 3HP in the homeless population compared to other regimens for treating LTBI?
29 What do you like best about using 3HP to treat homeless patients, in comparison to other treatment regimens?
30 What additional challenges do you think other health care providers or health facilities might face in adopting the use of 3HP in their health care setting?
31 Based on your experiences, do you feel that 3HP should be or should not be used in the homeless patient population? Please explain why.

## Supporting information

S1 Table Contact list for 3HP sites. (XLSX)
